# Odor perception and symptoms during acrolein exposure in individuals with and without building-related symptoms

**DOI:** 10.1038/s41598-022-12370-7

**Published:** 2022-05-17

**Authors:** Eva Palmquist, Anna-Sara Claeson

**Affiliations:** 1 Department of Psychology, Umeå University, 901 87 Umeå, Sweden; 2 Present Address: Department of Food, Nutrition and Culinary Science, Umeå University, 901 87 Umeå, Sweden

**Keywords:** Occupational health, Risk factors

## Abstract

Building-related symptoms (BRS) is a significant work-related and public health problem, characterized by non-specific symptoms occurring in a particular building. The cause of BRS is unknown, but certain reactive compounds are suggested risk factors. The aim of this controlled exposure study was to investigate whether BRS cases report more odor annoyance and symptoms and show altered autonomous nervous system (ANS) response during exposure to the reactive aldehyde, acrolein in comparison with referents. Individuals with BRS (n = 18) and referents (n = 14) took part in two exposure sessions (80 min). One session contained heptane alone, and the other heptane and acrolein. Perceived odor annoyance; eye, nose, and throat symptoms; and ANS response were measured continuously. BRS cases did not experience more odor annoyance; eye, nose, and throat symptoms; or altered ANS response in comparison with referents during the exposures. Supplementary analyses revealed that BRS cases that also reported chemical intolerance perceived more symptoms than referents during acrolein exposure. Acrolein exposure at a concentration below previously reported sensory irritation detection thresholds is perceived as more irritating by a subgroup of BRS individuals compared with referents. The results of this study indicate that a subset of individuals with building related symptoms (BRS) has a lowered sensory irritation threshold towards acrolein exposure. Future guidelines on chemical exposures to acrolein should take time and individual sensitivity into account.

## Introduction

Building-related symptoms (BRS) is a condition characterized by the manifestation of non-specific symptoms that occur in a particular building. Common symptoms in BRS are irritation of the mucous membranes and the upper respiratory tract, skin-related symptoms, headache, concentration difficulties, and lethargy^1^. Severe BRS leads to substantial suffering, functional impairment, and reduced quality of life^2–4^. Environmental factors in the buildings that have been associated with BRS are air contamination, poor ventilation, water damage/dampness, high temperature, and humidity. The symptoms are sometimes attributed to chemicals in the building, such as emissions from mold or building material (e.g. carpet glue)^5^. In fact, the overlap between BRS and chemical intolerance (CI, i.e. idiopathic environmental intolerance/multiple chemical sensitivity) is extensive as nearly 60% of the BRS cases also report CI^6^. CI can be described as a condition characterized by the subjective experience of symptoms attributed to chemical exposure that most people regard as unproblematic^7^. Examples of chemical exposure that elicit symptoms among CI cases are exposure to perfumes, cleaning agents, and printing ink. Narratives of individuals experiencing symptoms from residing in buildings with indoor air problems tell that health improvement is initially experienced when the afflicted individuals leave the building in which symptoms have commenced^8^. However, soon enough these individuals experience hypersensitivity to a wide range of everyday chemicals as well as symptoms when residing in other specific buildings. An initial intolerance to a specific building that eventually develops into a more generalized and severe sensitivity to odors has been identified in earlier studies^9–11^. Hence, initial BRS might develop into CI or a combination of the two conditions.

Two recent studies indicate an association between chemical compounds and indoor air health problems^12,13^. Among other compounds, the reactive aldehydes acrolein and formaldehyde are often pointed out as risk factors for symptoms reported in indoor air^14^. Acrolein is formed by incomplete combustion of wood, plastics, and petroleum-based fuels and from cooking^1,12^. It is also formed from oxidation of other volatile organic compounds^15^. Acrolein is also commonly found at higher concentrations in newly built homes and in wood material often used in building construction^15,16^ and has been found to impact chronic health more severely than environmental tobacco smoke and radon^17^. Acrolein has been proposed to be a previously overlooked risk factor for asthma^18^ and is known to induce sensory irritation in a time-dependent manner at a concentration below previously reported detection levels^19,20^. Acrolein together with other similar compounds is therefore of relevance to study further in relation to health problems in indoor air. The concentration of acrolein in indoor air has been found to be close to or above its odor threshold (but usually below sensory irritation detection), which may negatively affect the perceived indoor air quality due to its acrid odor^21^. Exposure studies on odorous chemicals at concentrations above their odor threshold but below their sensory irritation threshold have shown elevated symptom reporting especially among asthmatics and people with allergic rhinitis and dermatitis^22^. The odor of the chemicals might change the perception of the indoor air quality and might possibly cause environmental worry, stress, and negative mood that may influence reporting of sensory irritation^23^. Hence, the reporting of sensory irritation is affected by a number of non-sensory factors.

Acrolein activates transient receptor potential ankyrin 1 (TRPA1) ion channels^24,25^, and binding of acrolein (or other irritants) to the TRPA1-receptor on trigeminal nociceptors gives rise to sensory irritation in the eyes, throat, and nose. Upon activation, TRP channels also control the release of immunomodulatory neuropeptides (e.g. substance P) that trigger an inflammatory response, i.e. neurogenic inflammation^25^. Hence, the TRP receptors provide a mechanism for linking low exposure of pungent chemicals to inflammatory symptoms^26^. Initial high-level chemical exposure and/or tissue damage might upregulate the TRP channel activity, thus increasing irritant responsiveness^27^. Acrolein is a reactive compound that in a previous study has been shown to elicit higher levels of sensory irritation in individuals with CI in comparison to referents^20^, and trigeminal sensitivity was suggested as a cause of the sensitivity to chemicals in general.

One general response to both physical and emotional stress is activation of the autonomic nervous system (ANS). Major methods to study ANS activity include breathing rate, heart rate variability (HRV), and electrodermal activity (EDA)^28^. Inhalation of irritants, such as acrolein, might lead to decreased breathing rate because activation of TRP receptors in the airways reduces respiratory frequency as a protective measure against further inhalation^27^. On the contrary, emotional stress has been associated with an increase in breathing rate^29^. Exposure to particular air pollutants as well as mental stress have been found to reduce HRV^30,31^. Additionally, orthostatic, physical, and cognitive stress have been associated with increased electrodermal activity, which is a measure of the changes in electrical conductance of the skin due to sweat production^32^.

There are few experimental studies or studies on controlled exposure to specific chemicals (or other potential trigger factors) in relation to BRs. Acrolein is a reactive compound found at concentrations in indoor air that could elicit sensory irritation. Its time-dependent activation together with its effect on individuals with CI make acrolein suitable when it comes to investigating the impact of reactive compounds in BRS. The aim of this controlled human exposure study was to examine whether masked exposure to acrolein is associated with more odor annoyance, symptom reports, and/or altered ANS activity in individuals with BRS in comparison with referents. Based on previous results on the overlap between CI and BRS, we also wanted to investigate the influence of CI on symptoms during exposure to acrolein.

## Method

### Participants

Thirty-seven non-smoking individuals with and without BRS were recruited from a list of participants that had taken part in a previous study and who had consented to take part in additional future studies^13^. Participants were also recruited through advertisements in the local newspaper as well as through billboard advertisements. Twenty-one of the participants answered ‘yes’ to the question “Do you consider yourself to experience or have experienced symptoms caused by poor indoor environment at your work?” and were considered as BRS cases. A normal sense of smell was considered as an inclusion criterion and was tested using the Connecticut Chemosensory Clinical Research Center Threshold Test^33^. Dilution step 6 was used as the cut-off (*n-*butanol 0.44% (v/v); 336 ppm). All participants fulfilled the criteria. Participants with a cold or observable signs of upper respiratory illness (e.g., runny nose, sore throat, cough, etc.) at the time of testing were rescheduled.

Two of the BRS cases attended only one of the two chemical exposure sessions and were therefore excluded. One additional BRS case was excluded due to reporting very high levels of symptoms prior to exposure. Two of the referents reported experiencing symptoms from odorous/pungent chemicals from, for example, perfume or cleaning agents and not just restricted to “sick buildings”. These were excluded from the referent group because they could be considered as cases of CI, which in a previous study have been shown to report greater sensory irritation in the eyes, nose, and throat in comparison with referents^20^. This resulted in a final sample of 32 participants (18 BRS cases and 14 referents). Among those with BRS 8 reported to also have CI (they answered “yes” to the question “Are you experiencing symptoms from odorous/pungent chemicals (not limited to certain buildings), such as perfumes and cleaning agents, in doses that you were not getting symptoms from before or that you believe most other people are not getting symptoms from?”).

Prior to the first exposure session participants filled in a questionnaire consisting of demographic inquiries, the Perceived Stress Scale consisting of ten questions (PSS-10)^34,35^, the anxiety and depression subscales of The Hospital Anxiety and Depression Scale (HADS-A and HADS-D)^36^, the Shirom–Melamed Burnout Questionnaire (SMBQ)^37^, and the Chemical Sensitivity Scale for Sensory Hyperreactivity (CSS-SHR)^38^, which is a measure of affective reactions and behavioral disruptions by exposure to odorous/pungent chemicals. The questionnaire also included a list of symptoms commonly reported in indoor air. The participants rated each symptom on a 3-point scale (0 = No, never, 1 = Yes, sometimes, 2 = Yes, often) according to if they had experienced the symptom during the last three months. Symptoms were clustered into the following symptom groups: airway (9 symptoms), eyes and skin (6 symptoms), cardiac, nausea, and dizziness (4 symptoms), gastrointestinal and head related (5 symptoms), and cognitive and affective (9 symptoms), and sum totals for each symptom group were calculated. The sum totals of each symptom group and global values of each scale used were transformed into percentages of the maximum possible score.

Participants’ characteristics are presented in Table 1. Participants with BRS were older (t(30) = −2.34, *p* = 0.026) and reported more airway (t(30) = −3.47, *p* = 0.002) and eye and skin symptoms (t(30) = −2.88, *p* = 0.007) than referents. However, there are no consistent evidence for an association between age and BRS^39^. This difference was therefore not accounted for in the analysis. There was also a significant difference between groups regarding scores on the CSS-SHR (F(1, 30) = 3.75, *p* = 0.035) for which the BRS + CI group reported higher scores than referents (*p* = 0.036).

**Table 1:** Characteristics of the participants.

	Referents (n = 14)	BRI cases (n = 18)		
			BRI only (n = 10)	BRI + CI (n = 8)
Age: m(SD)	**36.9 (12.0)**	**46.2 (10.5)**	**47.1 (12.5)**	**45.1 (8.08)**
Female: n (%)	11 (78.6)	15 (83.3)	7 (70.0)	8 (100)
**Highest level of education: n (%)**				
Comprehensive school	0 (0.0)	1 (3.1)	1 (10.0)	0 (0.0)
Upper secondary school	1 (7.1)	5 (18.8)	1 (10.0)	4 (50.0)
University	13 (92.9)	12 (78.1)	8 (80.0)	4 (50.0)
**General health: n (%)**				
Excellent	1 (7.1)	0 (0.0)	0 (0.0)	0 (0.0)
Very good	0 (0.0)	1 (5.6)	0 (0.0)	1 (12.5)
Good	5 (35.7)	10 (55.6)	4 (40.0)	6 (75.0)
Fairly poor	6 (42.9)	5 (27.8)	4 (40.0)	1 (12.5)
Poor	2 (14.3)	2 (11.1)	2 (20.0)	0 (0.0)
SMBQ: m(SD)	36.6 (6.94)	33.9 (7.56)	31.9 (4.94)	36.4 (9.70)
PSS-10: m(SD)	35.5 (17.4)	30.1 (15.9)	28.0 (18.9)	32.8 (11.8)
HADS-D: m(SD)	6.46 (6.89)	7.67 (9.26)	7.62 (11.0)	7.74 (7.17)
HADS-A: m(SD)	14.3 (10.4)	9.79 (10.1)	8.10 (11.0)	11.9 (9.18)
CSS-SHR: m(SD)	47.9 (17.9)	61.1 (23.5)	52.4 (17.9)	**72.0 (26.2)**
**Symptoms: m(SD)**				
Airway	**17.5 (10.4)**	**42.3 (25.1)**	**33.3 (17.6)**	**53.5 (29.5)**
Eyes and skin	**13.7 (17.5)**	**43.5 (35.4)**	**31.7 (34.4)**	**58.3 (32.7)**
Cardiac, nausea and dizziness	5.36 (10.6)	11.0 (19.2)	2.78 (5.51)	20.3 (24.9)
Gastrointestinal and head related	25.71 (23.1)	28.89 (26.1)	13.0 (14.2)	48.8 (24.2)
Cognitive and affective	21.8 (21.4)	18.5 (16.3)	12.2 (14.3)	26.4 (15.9)

Significant values are in bold.

SMBQ = Shirom Melamed Burnout Questionnaire, PSS-10 = Perceived Stress Scale of ten questions, HADS-D = Hospital Anxiety and Depression Scale-Depression subscale, HADS-A = Hospital Anxiety and Depression Scale-Anxiety subscale, CSS-SHR = Chemical Sensitivity Scale for Sensory Hyperreactivity.

This study was approved by the Umeå Regional Ethics Board (Dnr: 2017/427-31) and was conducted in accordance with the Declaration of Helsinki. Prior to exposure the participants were informed that the exposure might lead to a slight irritation of the mucous membranes of the eyes, nose, and throat, and informed consent was obtained.

### Procedure

The participants attended two separate 80-min exposure sessions on separate days (at least two days apart). In one of the sessions, participants were exposed to a mixture of acrolein and heptane (i.e. masked acrolein exposure), whereas they were exposed to heptane alone at the other session. The order of the exposure sessions was balanced across participants and groups, i.e. half of the BRS cases and half of the referents were exposed to masked acrolein first, whereas the other half of the two groups were exposed to heptane alone first. The effects of exposure were investigated in a single-blinded manner because the participants were blinded to the exposure condition. Every five minutes (16 times) during every exposure the participants rated their perceived eye, nose, and throat symptoms as well as perceived odor intensity, and negative valence of the odor was rated using the Borg CR-100 scale^40^. Three times during each exposure (~ 25 min apart) the participants additionally reported skin, respiratory, concentration, dizziness, fatigue, nausea, and head symptoms using the Borg CR-100 scale. The mean of these ten symptom was calculated and used as a composite score in the statistical analysis. Additionally, all of these measures were also reported before (pre-test) and after (post-test) each exposure. During the whole exposure, ANS recordings of heart rate variability, electrodermal activity, and breathing rate were collected.

### Chemical exposure

Exposure occurred in a glass-walled exposure chamber (2.0 m × 0.9 m × 1.5 m). The mean temperature during exposure was 21.8 ± 1 °C and the mean relative humidity was 19.3 ± 3%. There were no differences in either temperature or relative humidity between the exposure conditions or between the groups (p-values between 0.19 and 0.92). Carbon-filtered air entered the chamber through an inlet close to the floor and exited through the ceiling. The air exchange rate was set to 7.5 air changes/h (approximately 330L/min). A metered amount of stimulus material was continuously vaporized through a nebulizer entering the air stream at a flow rate of 4 L/min.

The stimulus material consisted of acrolein (CAS: 188 29-55-5; Sigma Aldrich Co., St. Louis, MO, USA) and heptane (CAS: 142-82-5; Thermo Fisher Scientific, Waltham, MA, USA). The concentration of acrolein during both exposures was 0.05 ± 0.08 mg/m^3^, which was below previously reported sensory irritation threshold values (i.e. 0.11–1.2 mg/m^3^)^19,41–44^. Heptane was used as a solvent to dilute acrolein and also as an olfactory masking agent for acrolein in one of the exposure sessions and presented alone in the other exposure session thus serving as a control condition. The concentration of heptane during the control exposure was 10.3 ± 1.3 mg/m^3^ and during the masked acrolein exposure was 10.9 ± 1.7 mg/m^3^. An independent-samples *t*-test showed that the measured concentrations of heptane did not differ significantly between the two exposure conditions (*p* = 0.88).

### ANS recordings

Electrocardiogram (ECG) and breathing rate were measured with a Biopac MP100 system. Breathing rate was recorded by a chest strap that recorded changes in thoracic circumference caused by respiratory movements. It was recorded at 1000 Hz and filtered offline with a 0.05–1 Hz band-pass filter. Mean breathing rate was extracted from the waveform as 16 values based on 5-min blocks. The ECG was collected by attaching disposable electrodes to the non-dominant wrist and ankle, and it was performed at 1000 Hz and filtered offline with a 0.1–35 Hz band-pass filter. The root mean square of the normal-to-normal heartbeat interval difference (RMSSD) and the mean standard deviation of the normal to normal pulse intervals (SDNN), which are both measures of heart rate variability (HRV), were extracted into sixteen 5-min segments by using Kubios 2.1. Motion artifacts were removed manually after visual inspection, and the software’s algorithm was then used to correct for uncorrected or missing R peaks. Electrodermal activity (EDA) was recorded at 1000 Hz with reusable Ag–AgCl electrodes coated with electrode gel, which were fixed to the distal phalanges of the index and middle fingers with adhesive tape. Data were filtered offline with a 1 Hz low-pass filter, and phasic skin conductance responses were subsequently removed by subtracting duplicate 0.05 Hz high-pass-filtered waveforms from the original waveforms. Based on blocks of 5 min each (16 values for each exposure), the mean skin conductance level (SCL) was extracted as a measure of tonic EDA. Calculations of EDA and breathing rate data were performed in AcqKnowledge 4.2 (BIOPAC Systems, Inc.).

### Statistical analyses

Ratings of perceived odor intensity, odor valence, and symptoms were analyzed with two series of either 4 × 2 × 2 or 4 × 2 × 3 ANOVAs. Time (4 time points) and Exposure (acrolein and heptane, heptane only) were set as within-subject factors and Group (2 groups: BRS, referents or 3 groups: BRS + CI, BRS, referents) was set as a between-subjects factor.

Separate two-way mixed ANOVAs were conducted with Time (16 measures) as the within-subject factor, Group (2 groups: referents/BRS: referents/BRS/BRS + CI, respectively) as the between-subjects factor, and with eyes, nose, and throat symptoms as the dependent variable. Difference scores for each time point (the value at time t minus the pre-test value) were calculated for each dependent symptom variable to account for variances in the reported pre-test symptoms. Additionally, the ANS measurements: breathing rate, EDA, and HRV (RMSSD and SDNN) during exposure were used as separate dependent variables. These analyses were performed separately for the two exposure conditions of masked acrolein and heptane alone. Analyses were performed in SPSS v.26, and the α-level was set to 0.05. The Greenhouse–Geisser correction was applied to the analyses in which the assumption of sphericity was violated.

## Results

**Figure 1:**
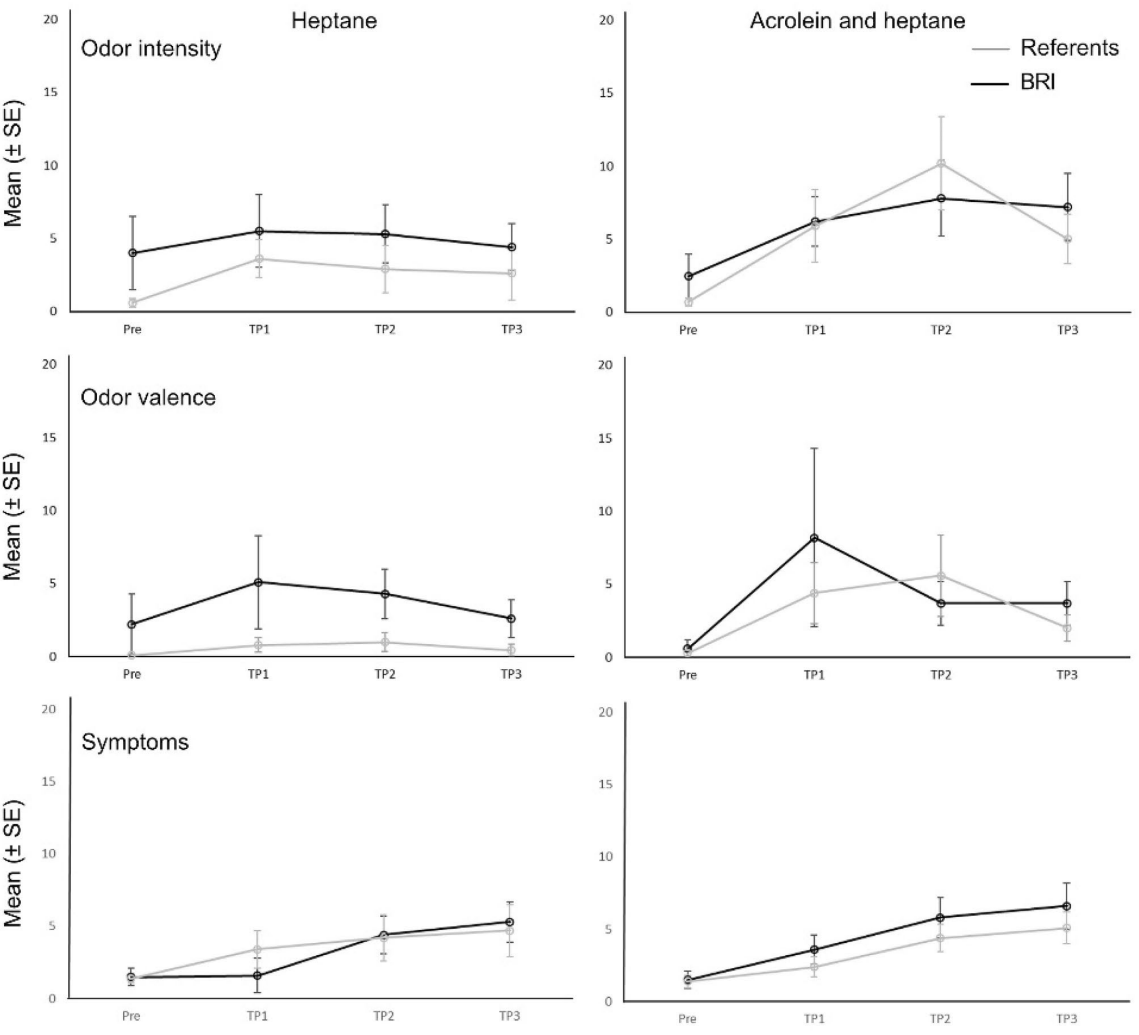
The mean ratings (Mean ± SE) of perceived odor intensity, valence, and ratings of symptoms for the referents and the group with building related intolerance, at four time points during exposure to heptane alone and acrolein and heptane.

The mean ratings of perceived odor intensity, valence, and ratings of symptoms for the referents and the BRS group at the four time points are presented in Fig. 1, and the results from the 4 × 2 × 2 (Time × Exposure × Group) ANOVAs are shown in detail in Table 2. No significant main effect of exposure or group or exposure × group interaction was identified.

**Table 2:** Results from the three-way ANOVAs for odor intensity, valence, and symptoms (F-values and, if statistically significant, ηp^2^ values within brackets) with two groups (Referents/BRS).

	Odor intensity			Odor valence			Symptoms		
	F	p-value	ηp^2^	F	p-value	ηp^2^	F	p-value	ηp^2^
Exposure (E)	2.57	0.120	0.08	1.40	0.246	0.05	0.17	0.688	0.01
Group (G)	0.95	0.339	0.03	1.29	0.266	0.04	0.31	0.585	0.10
Time (T)	**10.07**	**0.001**	**0.26**	2.70	0.09	0.09	**19.76**	** < 0.001**	**0.40**
E × G	0.55	0.466	0.19	0.59	0.449	0.02	0.29	0.597	0.01
E × T	1.76	0.162	0.06	0.66	0.463	0.02	1.03	0.374	0.03
T × G	0.37	0.652	0.01	0.48	0.588	0.02	0.30	0.667	0.01
E × T × G	0.37	0.773	0.01	0.37	0.607	0.01	0.22	0.842	0.01

Significant values are in bold.

**Figure 2:**
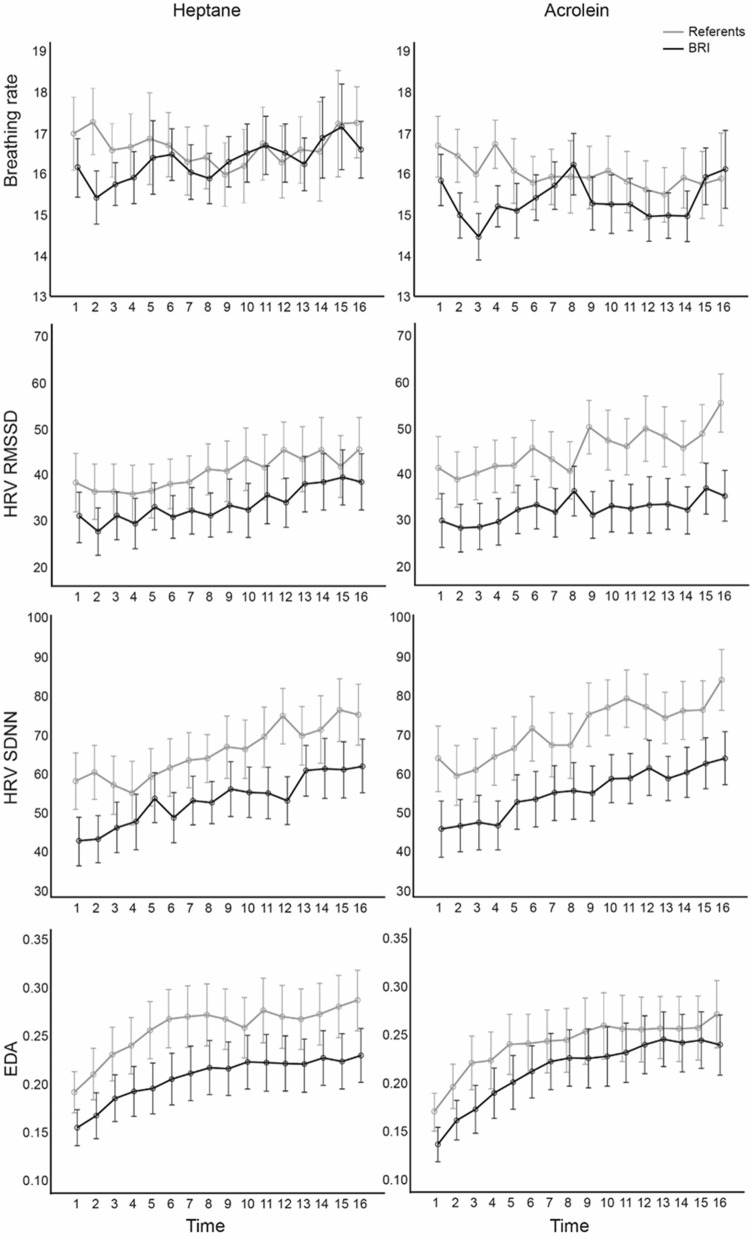
Estimated marginal means of breathing rate, RMSSD and SDNN measures of heart rate variability and electrodermal activity (EDA) over time in BRS cases and referents when exposed to Heptane and masked Acrolein.

Results from the mixed two-way ANOVAs with time (16 measures) as the within-subject factor, BRS as the between-subjects factor, and with ANS activity (breathing rate, HRV SDNN, HRV RMSSD, and EDA) as the dependent variable are presented in Fig. 2 and Table 3. There were no significant main effects of BRS on breathing rate, EDA, or any of the HRV measures. However, there were significant main effects of time on the HRV measures and on EDA, indicating that participants’ HRV and EDA increased over time. No significant main effect of time on breathing rate was found. There were no significant interaction effects between the effect of time and BRS, thus the effect of time on ANS activity was not different in BRS cases compared to referents.

**Table 3:** Results from the two-way mixed ANOVAs with Time (16 measuring points) as the within-subjects variable and Group (BRS/referents) as the between-subjects variable and with measures of autonomic nervous system response as the dependent variables in the heptane alone and masked acrolein exposure conditions.

Dependent variables	Independent variables	Heptane alone			Masked acrolein		
		F	*p*-value	ηp^2^	F	*p*-value	ηp^2^
Breathing rate	Group (G)	0.15	0.699	0.006	0.66	0.425	0.024
	Time (T)	0.69	0.636	0.026	0.89	0.504	0.032
	T × G	0.62	0.686	0.023	0.80	0.572	0.029
HRV SDNN	Group (G)	1.83	0.191	0.080	2.87	0.105	0.120
	Time (T)	**9.28**	** < 0.001**	**0.306**	**10.04**	** < 0.001**	**0.324**
	T × G	0.942	0.468	0.043	0.53	0.804	0.025
HRV RMSSD	Group (G)	0.79	0.383	0.036	2.90	0.104	0.121
	Time (T)	**5.99**	** < 0.001**	**0.222**	**5.48**	** < 0.001**	**0.207**
	T × G	0.84	0.538	0.038	1.88	0.089	0.082
EDA mean	Group (G)	1.63	0.212	0.051	0.46	0.503	0.017
	Time (T)	**17.89**	** < 0.001**	**0.373**	**23.70**	** < 0.001**	**0.467**
	T × G	0.512	0.658	0.017	0.77	0.490	0.028

Significant values are in bold.

**Figure 3:**
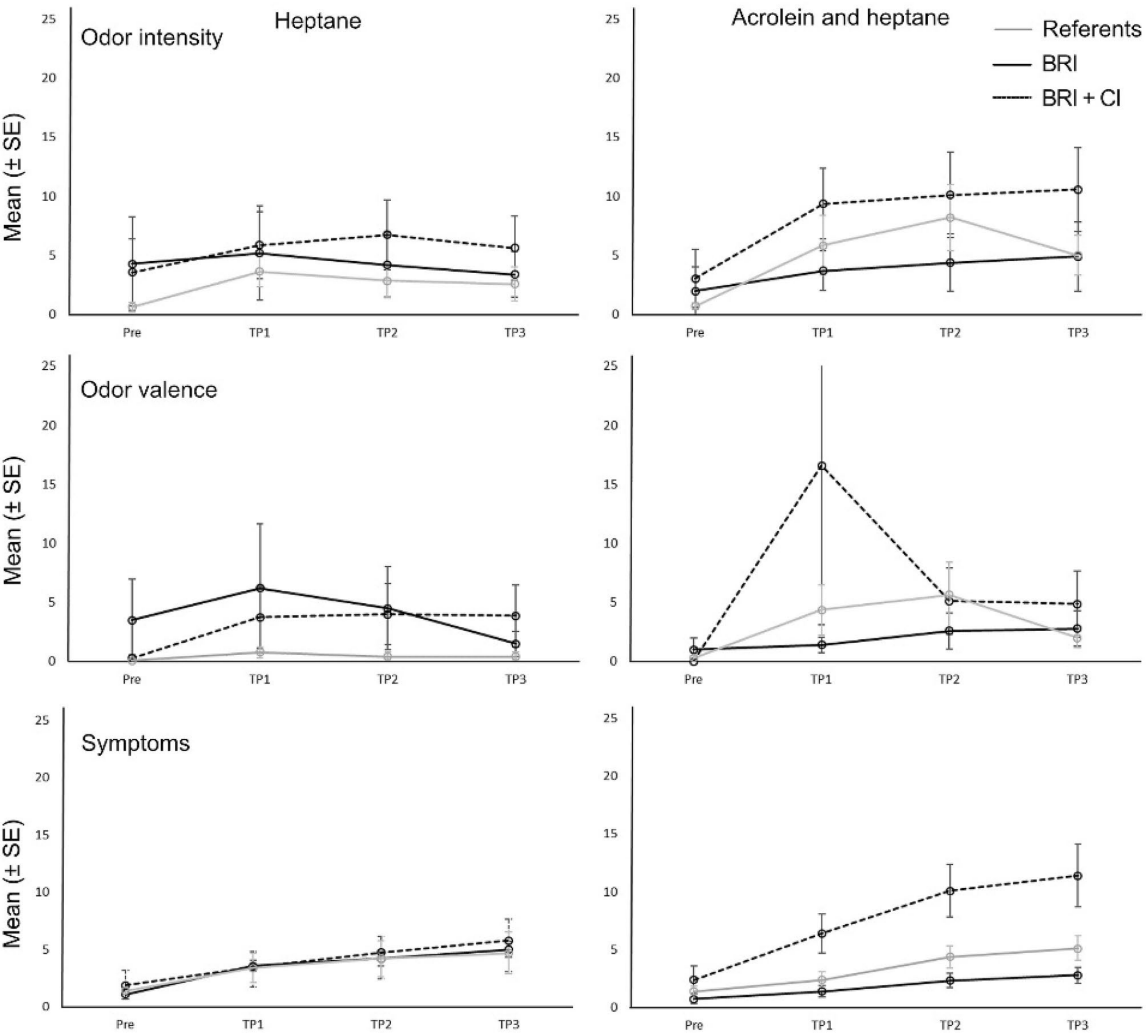
The mean ratings (Mean ± SE) of perceived odor intensity, valence, and ratings of symptoms for the referents and the two sub groups with building related intolerance (BRS and BRS + CI) at the four time points.

The role of CI in BRS was investigated, and a 4 × 2 × 2 ANOVA on the mean rating of the ten symptoms revealed an Exposure × Group interaction (F(2, 29) = 5,22, p = 0.012), and all results are shown in detail in Table 4. The mean ratings of perceived odor intensity, valence, and ratings of symptoms for the referents and the two BRS groups (BRS and BRS + CI) at the four time points are presented in Fig. 3.

**Table 4:** Results from the three-way ANOVAs for odor intensity and valence and symptoms (F-values and, if statistically significant, ηp^2^ values within brackets) with three groups (Referents/BRS/BRS + CI).

	Odor intensity			Odor valence			Symptoms		
	F	p-value	ηp^2^	F	p-value	ηp^2^	F	p-value	ηp^2^
Exposure (E)	2.51	0.125	0.09	1.45	0.238	0.05	0.93	0.344	0.03
Group (G)	1.78	0.187	0.12	1.14	0.335	0.08	2.19	0.130	0.13
Time (T)	**10.75**	**0.000**	**0.29**	**4.01**	**0.032**	**0.13**	**23.0**	**0.000**	**0.44**
E × G	1.12	0.342	0.08	1.83	0.180	0.12	**5.22**	**0.012**	**0.27**
E × T	1.52	0.216	0.05	1.00	0.394	0.04	1.51	0.223	0.05
T × G	1.53	0.178	0.10	1.29	0.272	0.09	1.36	0.267	0.09
E × T × G	0.27	0.906	0.02	1.39	0.263	0.09	2.11	0.072	0.13

Significant values are in bold.

**Figure 4:**
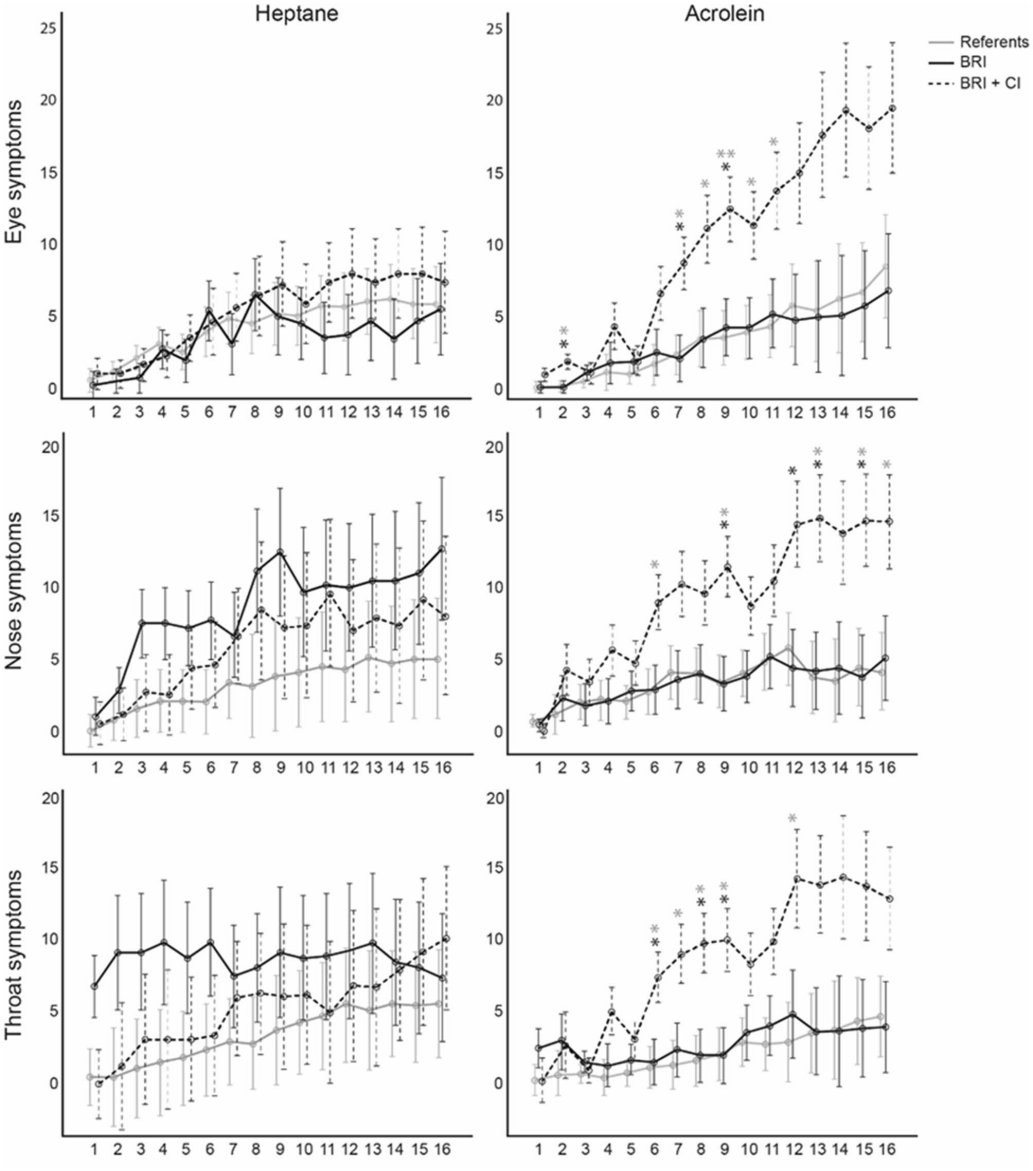
Estimated marginal means of difference scores of sensory irritation in the eyes, nose and throat over time in BRS + CI cases, BRS cases and referents when exposed to masked Acrolein and Heptane. Grey (BRS + CI vs. referents) and black (BRS + CI vs. BRS) asterisks refer to parameter estimates (*p < 0.05, **p < 0.01).

Separate detailed analyses were performed for the three most prominent symptoms of eye, nose, and throat irritation. Two-way mixed ANOVAs were performed separately for masked acrolein and heptane with the within-subject factors of Time (16 measures) and Group (referents, BRS cases, BRS + CI cases) and with the between-subject factors of eye, nose, and throat symptoms as the dependent variables. Bonferroni post-hoc analyses were used to determine variations between groups. Results are presented in Table 5 and Fig. 4. There were significant main effects of time on perceived eye, nose, and throat symptoms in both the heptane alone and masked acrolein conditions. There were also significant main effects of group (referents/BRS/BRS + CI) on all three symptoms in the masked acrolein condition but not in the heptane-alone condition. Post hoc analyses revealed that the BRS + CI group reported significantly more eye symptoms than both referents (*p* = 0.015) and the BRS group (*p* = 0.025), significantly more nose symptoms than referents (*p* = 0.026) and the BRS group (*p* = 0.050), and significantly more throat symptoms than referents (*p* = 0.043) but not more than the BRS group (*p* = 0.121).

**Table 5:** Results from the two-way mixed ANOVAs with Time (16 measuring points) as the within-subjects variable and Group (referents/BRS cases/BRS + CI cases) as the between-subjects variable and reported symptoms as the dependent variables in the heptane alone and masked acrolein exposure conditions.

Dependent variables	Independent variables	Heptane alone			Masked acrolein		
		F	*p*-value	ηp^2^	F	*p*-value	ηp^2^
Eye symptoms	Group (G)	0.19	0.872	0.013	**5.42**	**0.010**	**0.272**
	Time (T)	**6.78**	**0.002**	**0.189**	**11.10**	** < 0.001**	**0.277**
	T × G	0.47	0.775	0.031	1.63	0.193	0.101
Nose symptoms	Group (G)	0.72	0.493	0.048	**4.55**	**0.019**	**0.239**
	Time (T)	**6.81**	**0.003**	**0.190**	**7.21**	**0.012**	**0.199**
	T × G	0.58	0.669	0.038	1.71	0.153	0.105
Throat symptoms	Group (G)	0.54	0.588	0.036	**3.68**	**0.038**	**0.202**
	Time (T)	**3.86**	**0.026**	**0.117**	**6.75**	**0.003**	**0.189**
	T × G	1.36	0.260	0.085	1.89	0.132	0.115

Significant values are in bold.

Corresponding two-way mixed ANOVAs were also performed with the within-subjects factors of Time (16 measures) and Group (referents, BRS cases, BRS + CI cases) as the between-subjects factors and the measures of ANS activity (breathing rate, HRV SDNN, HRV RMSSD, and EDA) as the dependent variables. Results are presented in Table 5 and Fig. 4. As for the ANOVAs with the between-subjects factor consisting of two levels, there were only significant main effects of time on HRV SDNN, HRV RMSSD, and EDA in both the heptane alone and masked acrolein conditions. No significant main effect of group (referents/BRS/BRS + CI) nor any significant interaction effects on any of the dependent variables were found in either exposure condition (Table 6).

**Table 6:** Results from the two-way mixed ANOVAs with Time (16 measuring points) as the within-subjects variable and Group (referents/BRS cases/BRS + CI cases) as the between-subjects variable and with autonomic nervous system responses as the dependent variables in the heptane alone and masked acrolein exposure conditions.

Dependent variables	Independent variables	Heptane alone			Masked acrolein		
		F	*p*-value	ηp^2^	F	*p*-value	ηp^2^
Breathing rate	Group (G)	0.87	0.430	0.065	1.38	0.270	0.096
	Time (T)	0.93	0.461	0.036	1.24	0.286	0.046
	T × G	1.35	0.212	0.098	0.87	0.582	0.063
HRV SDNN	Group (G)	0.98	0.392	0.089	1.40	0.269	0.123
	Time (T)	**7.78**	** < 0.001**	**0.280**	**9.05**	** < 0.001**	**0.311**
	T × G	0.94	0.508	0.086	0.54	0.898	0.052
HRV RMSSD	Group (G)	0.44	0.652	0.042	1.38	0.275	0.121
	Time (T)	**5.94**	** < 0.001**	**0.229**	**4.37**	**0.001**	**0.179**
	T × G	0.625	0.806	0.059	1.66	0.088	0.142
EDA mean	Group (G)	1.01	0.376	0.065	0.33	0.725	0.024
	Time (T)	**15.62**	** < 0.001**	**0.350**	**23.29**	** < 0.001**	**0.472**
	T × G	0.63	0.694	0.041	0.48	0.785	0.036

Significant values are in bold.

## Discussion

No differences in perceived odor intensity, odor valence, symptoms, or ANS recordings between the whole group of BRS cases and referents or between the exposure conditions were identified. However, individuals reporting both BRS and CI differed from the referents as well as from the individuals with only BRS regarding the severity (Table 1) of reactions to the acrolein exposure (see Fig. 4).

The division of the BRS group into two separate groups was done on the basis of results from previous studies showing that about 60% of individuals with BRS also report having a more general sensitivity to chemicals (e.g. CI)^6^. In the current study, 18 participants reported having BRS, and of these eight also reported having CI. Data on severity (e.g. reported symptoms during the last 3 months) and chemical sensitivity (e.g. CSS, Table 1) indicated that this group was more affected by their condition than the group with only BRS, which implies a generalized and more severe condition, possibly developed over time as suggested in previous studies^8,11,45^. We therefore performed separate analyses for these three groups (Referents/BRS/BRS + CI) with the assumption that the combined group (BRS + CI) would react more strongly to the acrolein exposure than the other groups. As illustrated in Fig. 3, individuals with BRS + CI did indeed report more symptoms than the referents and the BRS group during exposure to acrolein. This result is in line with the results from the previous study on CI and acrolein exposure^20^. The groups did not differ in ratings of perceived odor intensity or valence.

The BRS + CI group reported more eye, nose, and throat symptoms than referents in the masked acrolein condition but not in the heptane-alone condition (Fig. 4). Even though these results should be interpreted with caution due to the low number of participants per group, the results are interesting. First of all, there was an increase in symptom reports after about 30–35 min of exposure (time points 6 and 7 in Fig. 4), which indicates a time dependence for sensory irritation, possibly due to interaction with the TRPA1-receptor^27^. This time dependence is of potential interest in indoor air settings and has been identified in earlier studies involving acrolein exposure^19,20,41^. In these studies, sensory irritation was detected after about 35 min in individuals with CI at a slightly higher acrolein concentration (0.07 mg/m^3^) than in the present study (0.05 mg/m^3^) and after about 7 min at a significantly higher concentration (0.35 mg/m^3^). Such time dependence has also been identified for other TRPA1 agonists, and it would be of interest to study the interaction effects from several TRPA1-agonists present in indoor air^46^.

Second, the perceived sensory irritation due to acrolein exposure near the detection threshold was only present in some of the participants (BRS + CI), a phenomenon also seen in previous studies involving acrolein exposure^19,41,44^. The increased sensitivity could be due to altered trigeminal reactivity, which is a commonly used theoretical explanation for CI^20,47,48^. The role of inflammation or oxidative stress that could have an effect on the sensitivity of the TRPA1 receptor needs to be further investigated; however, a previous study on inflammatory mediators measured before and after exposure to acrolein did not generate any results that supported the hypothesis about inflammation in CI^49^. Nevertheless, these results are of importance for individuals with BRS because they indicate that this group is not homogenous, which it is generally treated as being. As described by Schéle et al. (2019), initial BRS might develop into a combination of BRS and CI, and BRS + CI might be a more severe condition (implied by the symptom reports in Table 1) in which afflicted individuals react to a wider range of environmental exposures and are more hyperreactive to sensory stimuli. Alternatively, BRS cases constitute a heterogeneous group that experience symptoms from separate environmental exposures (e.g. microbial exposure, noise, vibration, temperature, odorous/pungent exposure), which plausibly makes them sensitive to that particular exposure and not necessarily to pungent chemicals. Hence, due to this heterogeneity it might have been a better approach to have screened participants for certain symptoms and symptom triggers at recruitment. The presence of CI and the severity of the affliction should also be taken into account in future exposure studies.

Acrolein has an odor that is generally described as unpleasant (even though the level of unpleasantness certainly varies between individuals) and heptane has a petroleum-like odor that many judge as unpleasant. Both BRS cases and referents perceived the odor as more intense and reported more symptoms over time in both exposure conditions. Further, HRV (RMSSD as well as SSDN) and EDA also increased over time. This indicates that the masking procedure was successful, and it is possible that the hedonic judgement and annoyance by the odors of the chemicals contributed to the rise of perceived symptoms for both BRS cases and referents regardless of exposure. The mechanism behind odors eliciting symptoms in the absence of irritation is not well understood, but hedonic judgements, annoyance, and negative expectations might be involved. The primary function of the olfactory system is life preservation as it guides food intake, warns about dangers such as fires and gas leaks, and modulates behavior^50^, and thus an unpleasant odor might be a signal of a potentially unsafe environment that should be avoided. However, there is large inter-individual variability in the hedonic judgements of the same odorous compound, and factors such as age, sex, cultural background, risk perception, and prior experience with the odor may affect such judgements^51^. Hedonic judgements have been found to affect odor annoyance^52^, which in turn is associated with symptom prevalence^52,53^. In this study, no difference in the perception of the odor was found between either of the groups, and the effects seen on symptoms in the BRS + CI group resembled the characteristic dependence on concentration commonly seen in sensory irritation (see Fig. 4).

This study has some limitations and one such limitation is that the case definition of BRS and CI solely rely on self-reports. However, the results of this study imply that future case definitions possibly also could include provocation challenges. Another limitation of this study, as already mentioned, is the low number of participants per group when splitting the BRS group into two (BRS + CI and BRS without CI), which means that the results have to be interpreted cautiously. An advantage of the present study is that it is one of few studies on controlled exposure to a specific chemical within the field of BRS research.

## Data Availability

The datasets used and/or analysed during the current study available from the corresponding author on reasonable request.
